# A Bird’s Eye View of Discard Reforms: Bird-Borne Cameras Reveal Seabird/Fishery Interactions

**DOI:** 10.1371/journal.pone.0057376

**Published:** 2013-03-06

**Authors:** Stephen C. Votier, Anthony Bicknell, Samantha L. Cox, Kylie L. Scales, Samantha C. Patrick

**Affiliations:** 1 Environment and Sustainability Institute, University of Exeter, Penryn, Cornwall, United Kingdom; 2 Marine Biology and Ecology Research Centre, Plymouth University, Plymouth, United Kingdom; 3 Plymouth Marine Laboratory, Prospect Place, The Hoe, Plymouth, United Kingdom; 4 Centre d’Etudes Biologiques de Chizé – CNRS, Villiers-en-Bois, France; Institute of Marine Research, Norway

## Abstract

Commercial capture fisheries produce huge quantities of offal, as well as undersized and unwanted catch in the form of discards. Declines in global catches and legislation to ban discarding will significantly reduce discards, but this subsidy supports a large scavenger community. Understanding the potential impact of declining discards for scavengers should feature in an eco-system based approach to fisheries management, but requires greater knowledge of scavenger/fishery interactions. Here we use bird-borne cameras, in tandem with GPS loggers, to provide a unique view of seabird/fishery interactions. 20,643 digital images (one min^−1^) from ten bird-borne cameras deployed on central place northern gannets *Morus bassanus* revealed that all birds photographed fishing vessels. These were large (>15 m) boats, with no small-scale vessels. Virtually all vessels were trawlers, and gannets were almost always accompanied by other scavenging birds. All individuals exhibited an Area-Restricted Search (ARS) during foraging, but only 42% of ARS were associated with fishing vessels, indicating much ‘natural’ foraging. The proportion of ARS behaviours associated with fishing boats were higher for males (81%) than females (30%), although the reasons for this are currently unclear. Our study illustrates that fisheries form a very important component of the prey-landscape for foraging gannets and that a discard ban, such as that proposed under reforms of the EU Common Fisheries Policy, may have a significant impact on gannet behaviour, particularly males. However, a continued reliance on ‘natural’ foraging suggests the ability to switch away from scavenging, but only if there is sufficient food to meet their needs in the absence of a discard subsidy.

## Introduction

Globally, commercial capture fisheries generate huge quantities of discards in the form of offal, unwanted or over-quota catch – during 1992–2001 an average of 7.3 million tonnes of fish were discarded each year [1]. This level of discarding is not sustainable and has also been shown to negatively impact ecosystem functioning and biodiversity [2], [3]. Yet despite this, discards represent a significant source of food for a large guild of scavenging seabirds. Provision of this novel and abundant food has led to changes in seabird movement patterns [4], breeding success [5], over-winter condition [6], population dynamics [7] and community composition [8]–[10], with populations of some discard-feeding generalist seabirds at historically high levels [3]. However, current levels of discarding are likely to decrease considerably as fishing practices change. As global catches decrease, and demand for protein increases, discard rates are likely to reduce significantly as a greater proportion of catches are retained [11]. Moreover, changes in fisheries management may fundamentally alter discard production. For instance, in the European Union (EU), forthcoming reforms to the Common Fisheries Policy (CFP) may lead to a complete ban on discarding altogether [2]. Although desirable, diminishing discards may have direct and indirect negative consequences for some seabird communities, which is of particular concern given that many seabird species are facing unprecedented rates of decline [12]. Understanding the impact of fisheries reforms is also important under an ecosystem approach to fisheries management but is hampered by our poor understanding of scavenging ecology.

The study of seabird/fishery interactions is logistically challenging. Historically, it has been conducted from boats, and this has provided much information on scavenging behaviour [13]. However, boat-based research fails to determine the reproductive status, sex or origin of scavengers, or whether scavengers also search for natural prey. Together this greatly limits the efficacy of boat-based approaches for determining the impact of discard reforms on seabirds. Diet reconstruction, either directly via prey remains or indirectly via elemental analysis, has also done much to improve our understanding of the importance of discards for seabirds [14]–[16]. However, it is not always possible to differentiate between those fish that are discards and those that are not, making it difficult to accurately quantify discard consumption [16]. The use of bio-logging devices such as GPS, geo-locators and immersion loggers, in tandem with spatial information on fisheries activity such as from vessel monitoring systems (VMS), has, thus far, been most effective in revealing the nature of overlap between seabirds and fisheries at meso [17] and sub-mesoscales [15], [18]. Nevertheless, even these approaches have a number of limitations: spatial overlap between seabirds and vessels does not necessarily mean interaction; vessel location data is normally gathered at a much coarser resolution than animal-borne tracking devices; gear-type information can be difficult to obtain; not all vessels are legally required to use vessel monitoring systems; and illegal, unreported and unregulated (IUU) fisheries may have significant ecological impacts but remain virtually impossible to monitor [19].

Bird-borne cameras offer a more tractable solution to this problem [20], [21]_ENREF_15. Such devices, with an appropriate duty cycle, can be used to record the presence/absence of fishing vessels during entire foraging trips. Used together with bird-borne GPS loggers, it is possible to reliably establish the extent to which searching occurs in the presence or absence of vessels. Although previous studies have assessed the extent to which tracked seabirds interact with fisheries [4], [15], they were unable to unequivocally exclude the presence of IUU fishing activity, or fisheries operating without using a Vessel Monitoring System (VMS).

Seabirds may respond to a discard ban in a number of ways. For generalist predators that feed facultatively on fishery waste, they may be able to switch to feed on smaller seabirds at colonies or at-sea, with potentially negative consequences [8]. However, piscivorous species are not able to switch in this manner, being constrained to forage for fish at sea. Therefore, these species may be particularly impacted by discard reforms if they have become too reliant on a prey landscape dominated by fishing vessels [4], [15], [18] – each a conspicuous and rewarding prey patch - at the expense of searching for naturally occurring prey. Therefore, key to knowing how pelagic seabirds will respond to discard reforms is to know whether they continue to search for both natural and fisheries derived prey.

Here we used a combination of miniaturised digital cameras and GPS loggers, recording at one-minute intervals, to study in detail the at-sea behaviour of chick-rearing northern gannets *Morus bassanus* (hereafter gannet) in relation to fishing boats. Gannets are large (∼3 km) wide-ranging piscivorous marine predators that feed on fishery discards [15], as well as a range of mesotrophic fish [15], [22], [23]. Using bird-borne cameras we quantify interactions between gannets and fishing vessels, test for sex effects and provide information on the size and gear type of fishing boats visited. In addition, we use GPS to examine fine-scale foraging strategies (using first-passage time (FPT) to detect Area Restricted Search (ARS) behaviour [24], [25]) in relation to fishing boats to test the hypothesis that gannets may be able to switch to natural foraging in the face of a discard ban.

## Methods

### Bird Sampling, Device Deployment and Ethical Statement

All fieldwork was conducted on Grassholm Island, Wales, UK (51° 43′ N, 05° 28′ W) during July 2011 under licence from Countryside Council for Wales and the British Trust for Ornithology. Birds were sexed using standard molecular techniques (Avian Biotech.com) from a blood sample taken under licence from the UK Home Office. We equipped 20 chick-rearing gannets with; (1) a 45 g digital camera (Perthold Engineering, Germany) with a fish-eye lens, attached facing backwards with Tesa© tape to the central tail feathers and (2) a 30 g GPS logger (iGotU GT-600, Mobile Technology), taped to feathers in the centre of the back. All birds were caught during changeover, to minimise time the chick was alone and ensure foraging trips began immediately following release. Total handling time did not exceed 15 minutes. Both devices were programmed to record information at one-minute intervals. We recovered 19 (95%) of the devices after 1 or 2 complete foraging trips and there were no significant differences in foraging trip duration (t-test with unequal variance: t_19.087_ = 1.507, p = 0.148) or foraging trip length (t-test with unequal variance: t_22.367_ = 0.910, p = 0.373) between 17 gannets with both a GPS and a camera and 17 control birds with a GPS only, tracked over the same period.

### Analysis Techniques

We first determined gear type and approximate vessel length by carefully examining photographs from the instrumented birds. This was done by the authors and by two professional fishermen currently operating in waters around SW Britain (where the gannets were foraging). Second, we used a combination of GPS tracks and photographs to determine how fishing vessels influenced gannet foraging behaviour. We used FPT [26] to identify ARS because initiation of this behaviour has been shown to be triggered by the detection and pursuit of prey in gannets [24]. FPT was calculated at interpolated intervals of 500 m along all daylight sections of foraging trips, excluding time spent at the colony, using standard techniques in the adehabitatLT package in R [27]. ARS zones were identified using an approach based on Lavielle segmentation within the adehabitatLT package. We plotted all gannet foraging tracks and ARS zones using ArcGIS (ArcMap v10. ESRI, Redlands, California) and compared these with fishing vessel locations matched with a time stamp.

## Results

### Device Retrieval

We recovered 19 (95%) of the birds with cameras and GPS, but only 10 of these had a set of images covering at least one complete foraging trip together with matching one-minute GPS fixes. Device failure arose because of electronic faults, water ingress or the lens becoming obscured by gannet plumage. Our dataset for analysis comprised 20,643 images, time-matched with GPS fixes, for 3 males and 6 females, plus one bird that could not be sexed.

### Encounters with Fishing Vessels

**Figure 1:**
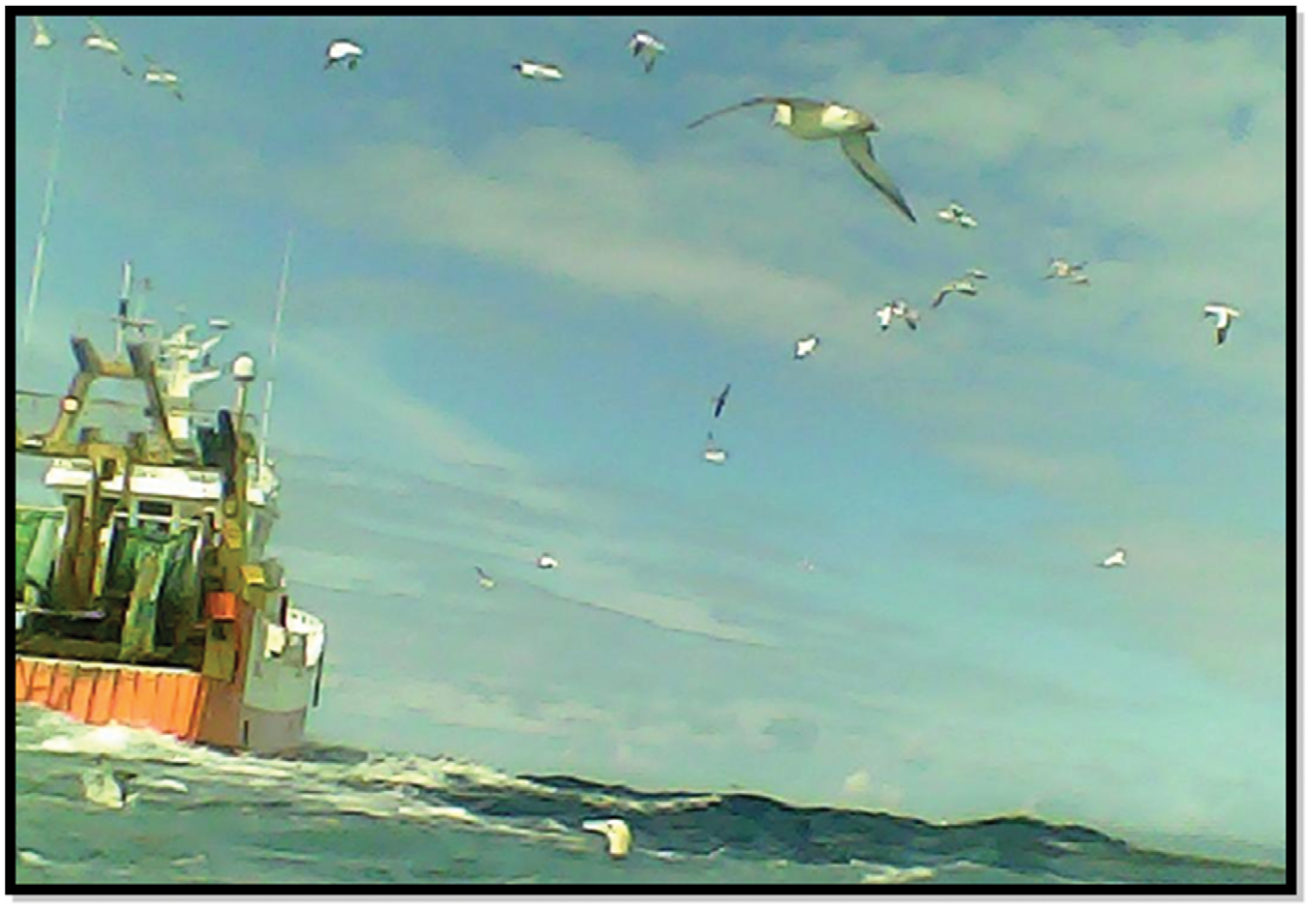
Bird-borne cameras reveal seabird/fishery interactions. All tracked gannets photographed fishing vessels, often in the company of large groups of other scavengers.

**Figure 2:**
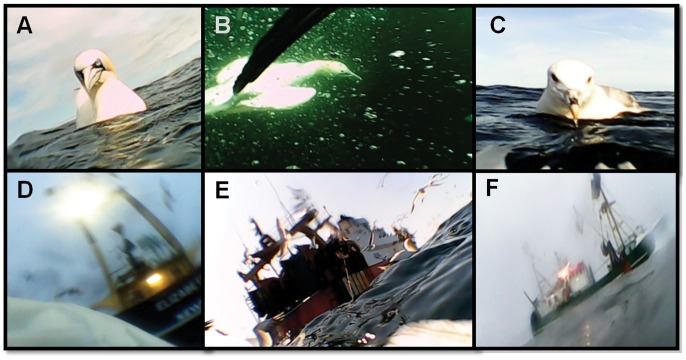
Bird-borne cameras reveal intra- and inter-specific interactions, and fishing vessel type. 93% of vessel encounters illustrated that northern gannets foraged with other birds, including conspecifics above (A) and below the water (B), as well as northern fulmars (*Fulmarus glacialis*) (C). Fishing vessels were visited during crepuscular foraging (D). The vast majority (95%) of fishing vessels encountered were trawlers, including stern trawlers (E) and beam trawlers (F).

All ten gannets photographed fishing vessels (Figures 1 & 2). There were a total of 28 fishing vessel encounters (interactions excluding repeat photographs of the same vessel) and the mean number of vessel encounters per foraging trip was 2.64 (±1.36). For 21 fishing vessels it was possible to identify gear type; there were 19 (90.5%) stern trawlers, 1 (4.8%) beam trawler and 1 (4.8%) gill netter (Figures 1 & 2). There were no artisanal boats; all were judged to be >15 m in length. Of the 28 fishing vessel encounters, 26 (92.9%) included associations with other scavenging seabirds, often in large multi-species groups (flocks included northern fulmars *Fulmarus glacialis*, large gulls *Larus spp*. and conspecifics) (Figures 1 & 2).

### Searching Behaviour

**Figure 3:**
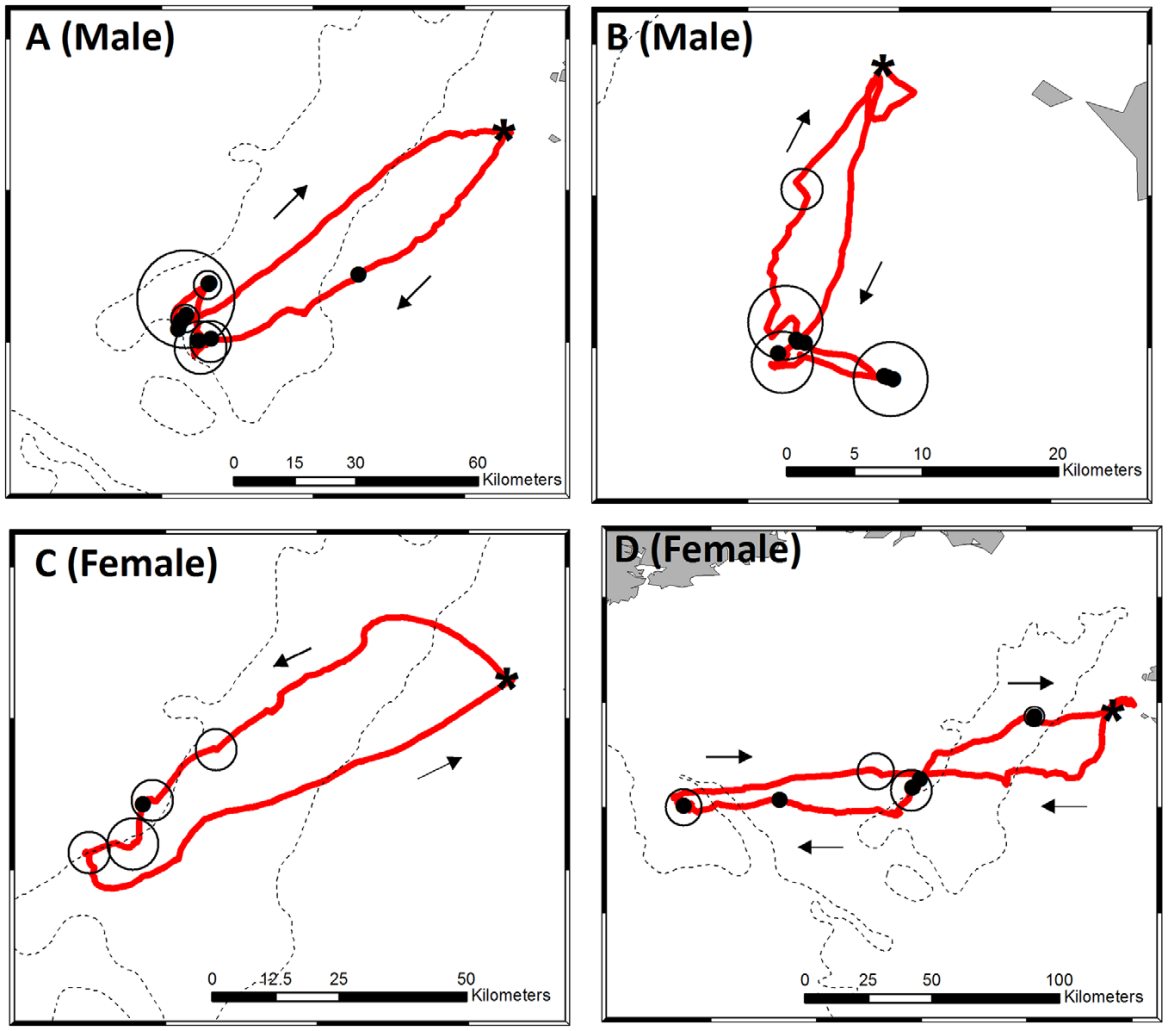
Bird-borne cameras and GPS reveal gannet search behaviours in relation to fishing activity. Figures show single foraging trips of four gannets reconstructed using fixes from GPS loggers. Open circles indicate location of area-restricted search (ARS) zones along the foraging track and are proportional to the scale of the ARS zone. Closed circles indicate the location of fishing vessels photographed by gannet-borne cameras. Arrows show the direction of travel and an asterix the location of the colony. Gannets showed a mixed foraging strategy, illustrated by ARS zones occurring with and without the fishing vessels. Males A & B foraged more at fishing boats compared with females C & D.

All individuals exhibited an Area-Restricted Search (ARS) during foraging trips (Figure S1), averaging 7.73 (±6.10) ARS events per trip. In relation to fishing activity, gannets typically showed a mixed foraging strategy; some ARS zones were associated with photographs of fishing vessels, while others were not (Figure 3). On average, across all birds, 42.2% of ARS zones were associated with photographs of fishing vessels while the remaining 57.8% were without. There were strong differences between the sexes in the proportion of ARS zones associated with fishing vessels –80.6% (n = 3) for males and 30.0% (n = 6) for females.

## Discussion

Our combined use of bird-borne cameras to photograph fishing boats and GPS loggers to reconstruct fine-scale foraging provides unique insights into seabird/fishery interactions. All ten of our tracked gannets photographed fishing vessels, indicating that scavenging is more common in this species than previously thought [15]. Based on these findings we might predict that gannets would be severely impacted as discards decline, but analysis of foraging behaviour reveal that most individuals showed a mixture of scavenging and ‘natural’ foraging. The implications of our findings in light of fisheries reform, as well as the results of using bird-borne cameras, are discussed below.

Although we cannot exclude the possibility that gannets encountered some vessels that were not photographed, this seems unlikely within ARS zones. Although our cameras faced backwards, they had a fish-eye lens ensuring a wide field of view, and because ARS behaviours are characterised by a high degree of turning and decreased flight speed, the probability of missing vessels in such areas is low. Furthermore, some boats were photographed during commuting flights (Figure 3A) indicating our approach has a high rate of vessel detection. Therefore, we are confident that our approach accurately characterises the presence/absence of fishing vessels in ARS zones.

The vast majority of photographs of fishing vessel encounters (93%) also showed other scavenging seabirds, sometimes in large numbers (Figures 1 & 2). Although mixed species flocks are a common feature of seabirds following fishing boats [13], our data is the first to quantify the frequency of this behaviour at the individual-level and highlights the significance of interference competition while scavenging. Moreover, local enhancement appears to be an important mechanism by which wide-ranging seabirds locate prey [28] and these mixed-species aggregations are likely to be an important sensory signal for species searching by sight. One consequence of eliminating discards therefor may be a reduction in the size and persistence of seabird foraging flocks, with potential implications for social facilitation and searching [29].

Analysis of ARS zones in relation to photographs of fishing boats revealed most gannets adopted a mixed foraging strategy - ARS zones occurred both in the presence and absence of fishing vessels (Figure 3 & S1). This suggests that, despite the importance of fisheries in the prey-landscape of gannets, they frequently use other non-fishery cues to locate prey. Maintaining this ‘natural’ search behaviour suggests the ability to adapt well if discards were to disappear altogether, although this would only be possible if there were sufficient food to meet seabirds’ nutritional needs in the absence of a discard subsidy [30]. Our work is not the first to show foraging flexibility in seabirds [23], but instead highlights that, from a behavioural perspective, scavenging is not necessarily an ecological dead-end.

Male gannets had a much higher proportion of ARS zones associated with fishing vessels compared with females (81% vs 30%). Although our sample sizes are very small (3 males and 6 females), these findings are consistent with previous work using stable isotopes, which suggest that male gannets feed more on discards than females [31]. The reasons for these gender-related differences are unclear, especially since gannets only show very slight sexual dimorphism [29]. However there may be sex-specific differences in competitive ability, nutritional requirements and/or parental roles [29]. Whatever the reason, these results suggest that male gannets may be impacted more by discard declines than females.

Some photographs of fishing boats enabled us to determine gear-type and approximate vessel size (Figure 1 & 2). Where we were able to establish gear-type, 93% of vessels were trawlers, which target demersal or mid-water fish. In the English Channel, Western approaches, Celtic and Irish seas (the main foraging areas used by gannets in this study), beam trawlers and otter trawlers are responsible for 90% of the 24,500 tonnes of discards produced during 2002–2005 [32]. Vessel photographs also showed that gannets foraged at large trawlers >15 m in length and no small artisanal boats. This finding is relevant for the use of VMS as a monitoring tool, since it is only a legal requirement for vessels >15 m.

At 45 g our cameras are currently only suitable for relatively large species of bird. If we assume that bird-borne devices should not exceed 3% of body mass (although we accept this is not the only consideration regarding potential device effects on birds [33]), this means these cameras could be used on birds ∼1550 g in weight. However, camera weight can be reduced further, by removing the fish-eye lens, to ∼32 g, enabling their use on smaller species (∼1100 g). Where body mass is appropriate for safe deployment, these (inexpensive) cameras could be used for bycatch mitigation research and potentially to monitor IUU fisheries, as well as for studying various aspects of searching behaviour such as information transfer and local enhancement.

In summary, we provide proof-of-concept that bird-borne cameras can be used to study seabird/fishery interactions. This could be particularly valuable in areas where seabird bycatch rates and discard consumption are high. The method also uniquely incorporates the birds’ colony of origin, sex and reproductive status; key information to assess the potential impact of discard declines. Understanding such wide-scale impacts is central to an eco-system approach to fisheries management in general, and reforms of the EU CFP in particular. Moreover, when used together with GPS loggers, this technique may be powerful in the battle against illegal, unreported and unregulated fishing activity, which is likely to produce significant amounts of bycatch and discards but remains difficult to study [19].

## Supporting Information

Figure S1 **GPS tracks showing foraging trips of all chick-rearing northern gannets fitted with bird-borne digital cameras from Grassholm, Wales 2011.** Open circles show ARS zones, closed circles the location of fishing vessels photographed by each bird and arrows show the direction of flight.
(DOCX)
