# Supplementary material for: A Bird’s Eye View of Discard Reforms: Bird-Borne Cameras Reveal Seabird/Fishery Interactions
Source: PLoS One. 2013 Mar 6;8(3):e57376. doi: 10.1371/journal.pone.0057376 (PMC3590202; doi:10.1371/journal.pone.0057376)
Supplement: Supplementary file 1 [file pone.0057376.s001.docx]

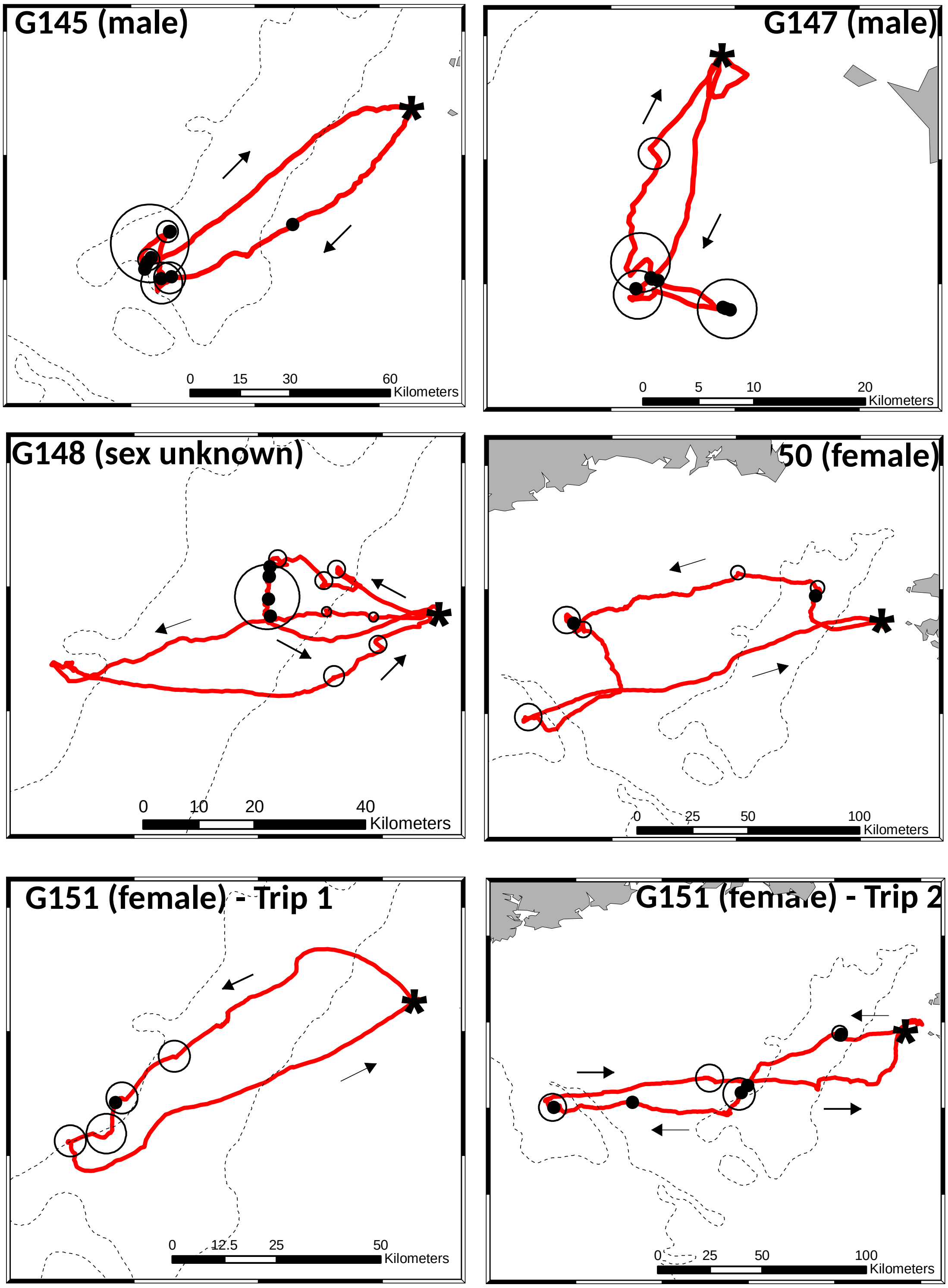
G145 (male)
0 15 30 60
Kilometers
G147 (male)
0 5 10 20
Kilometers
G148 (sex unknown)
0 10 20 40
Kilometers
50 (female)
0 25 50 100
Kilometers
G151 (female) - Trip 1
0 12.5 25 50
Kilometers
G151 (fem e) - Trip 2
0 25 50 100
Kilometers

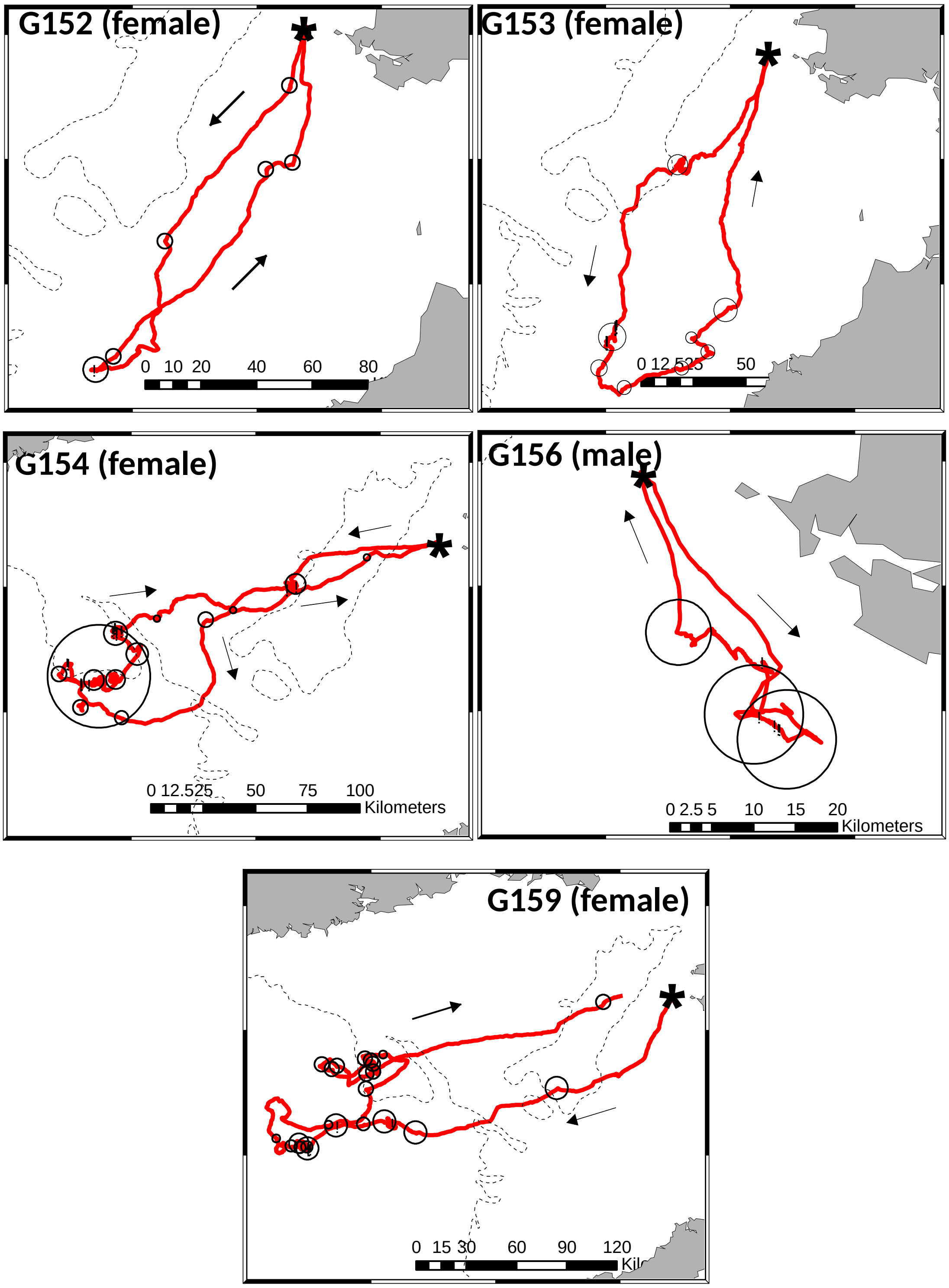
G152 (female)
0 10 20 40 60 80
G153 (female)
0 12.525 50
G154 (female)
0 12.525 50 75 100
Kilometers
G156 (male)
0 2.5 5 10 15 20
Kilometers
G159 (female)
0 15 30 60 90 120
